# Large language model-based multimodal system for detecting and grading ocular surface diseases from smartphone images

**DOI:** 10.3389/fcell.2025.1600202

**Published:** 2025-05-23

**Authors:** Zhongwen Li, Zhouqian Wang, Liheng Xiu, Pengyao Zhang, Wenfang Wang, Yangyang Wang, Gang Chen, Weihua Yang, Wei Chen

**Affiliations:** ^1^ Ningbo Key Laboratory of Medical Research on Blinding Eye Diseases, Ningbo Eye Institute, Ningbo Eye Hospital, Wenzhou Medical University, Ningbo, China; ^2^ National Clinical Research Center for Ocular Diseases, Eye Hospital, Wenzhou Medical University, Wenzhou, China; ^3^ Department of Ophthalmology, West China Second University Hospital, Sichuan University, Chengdu, China; ^4^ First People’s Hospital of Aksu, Aksu, China; ^5^ Shenzhen Eye Hospital, Shenzhen Eye Medical Center, Southern Medical University, Shenzhen, China

**Keywords:** ocular surface disease, large language model, multimodal model, keratitis, conjunctivitis, pterygium

## Abstract

**Background:**

The development of medical artificial intelligence (AI) models is primarily driven by the need to address healthcare resource scarcity, particularly in underserved regions. Proposing an affordable, accessible, interpretable, and automated AI system for non-clinical settings is crucial to expanding access to quality healthcare.

**Methods:**

This cross-sectional study developed the Multimodal Ocular Surface Assessment and Interpretation Copilot (MOSAIC) using three multimodal large language models: gpt-4-turbo, claude-3-opus, and gemini-1.5-pro-latest, for detecting three ocular surface diseases (OSDs) and grading keratitis and pterygium. A total of 375 smartphone-captured ocular surface images collected from 290 eyes were utilized to validate MOSAIC. The performance of MOSAIC was evaluated in both zero-shot and few-shot settings, with tasks including image quality control, OSD detection, analysis of the severity of keratitis, and pterygium grading. The interpretability of the system was also evaluated.

**Results:**

MOSAIC achieved 95.00% accuracy in image quality control, 86.96% in OSD detection, 88.33% in distinguishing mild from severe keratitis, and 66.67% in determining pterygium grades with five-shot settings. The performance significantly improved with the increasing learning shots (p < 0.01). The system attained high ROUGE-L F1 scores of 0.70–0.78, depicting its interpretable image comprehension capability.

**Conclusion:**

MOSAIC exhibited exceptional few-shot learning capabilities, achieving high accuracy in OSD management with minimal training examples. This system has significant potential for smartphone integration to enhance the accessibility and effectiveness of OSD detection and grading in resource-limited settings.

## Introduction

Ocular surface diseases (OSDs) significantly contribute to global eye health challenges (Burton et al., 2021). Several OSDs can lead to serious adverse consequences if not addressed timely. For instance, keratitis is a leading cause of corneal blindness and visual impairment worldwide (Stapleton, 2023). Conjunctivitis, a prevalent condition, imposes substantial economic and social burdens (Azari and Barney, 2013). Additionally, pterygium, one of the most common eye disorders, is associated with aesthetic concerns, irregular astigmatism, and decreased vision (Rezvan et al., 2018). Early detection and appropriate treatment of OSDs are crucial for preventing vision loss and preserving ocular health (Saaddine et al., 2003).

Unfortunately, access to specialized ophthalmic care is often limited, particularly in underserved regions, impeding timely diagnosis of OSDs (Resnikoff et al., 2012; Gupta et al., 2013). While portable devices have been employed in some studies to capture images in non-clinical settings, prompt responses from experienced experts remain indispensable (Caffery et al., 2019). Recent studies have leveraged artificial intelligence (AI) to develop efficient solutions for automated disease detection and management, aiming to mitigate the shortage of expert resources (Tan et al., 2023; Li et al., 2024)

Despite the significant advancements in AI, its integration into clinical practice faces several challenges. Firstly, although numerous studies have developed AI systems, patients are often unable to access them due to the lack of public availability and the persistent gap between research and product implementation (Closing the translation gap, 2025). Furthermore, most studies applying AI to analyze OSDs relied on anterior segment images captured by specialized devices such as the slit lamp, limiting the models’ applicability in remote and underserved regions (Zhang et al., 2023; Zhongwen et al., 2025). These significant obstacles contribute to the absence of an efficient and practical tool for detecting and managing OSDs.

To make medical AI services more accessible, we developed Multimodal Ocular Surface Assessment Intelligent Copilot (MOSAIC), a large language model-based AI system with extensible components, which included modularized agents for image quality control, OSD recognition, analysis of the severity of keratitis, and pterygium grading. MOSAIC is constructed by integrating publicly accessible multimodal large language models (MLLMs) with the strategies of prompt engineering and few-shot prompt learning. Prompt engineering and few-shot prompt learning have shown potential as effective methods in optimizing and adjusting large language models (Wang et al., 2024; Šuster et al., 2024). Based on MOSAIC, we established an automated pipeline for analyzing OSDs from smartphone images. To be specific, we first validated MOSAIC’s ability to monitor image quality, which is used to filter out poor-quality images and identify the reasons for their inadequacy. In addition, we assessed MOSAIC’s ability to detect keratitis, conjunctivitis, and pterygium using the Union Centers Smartphone Image (UCSI) dataset. Furthermore, we investigated MOSAIC’s ability to aid disease management by identifying mild-stage keratitis for early intervention and assessing pterygium severity to determine the optimal timing for surgery. Finally, we explored MOSAIC’s interpretability by assessing its image comprehension capability. This study demonstrated that MOSAIC offers great potential for detecting and grading OSDs in general populations within non-clinical settings.

## Methods

### Design of MOSAIC

**FIGURE 1:**
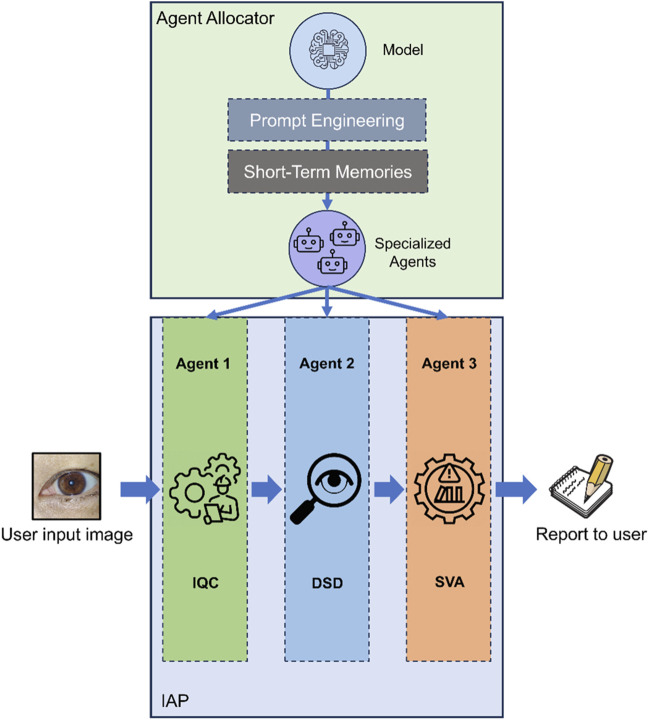
Design and architecture of MOSAIC. MOSAIC was constructed with several components to keep its extendibility. The agent allocator acts as a router to create specific agents for sub-tasks in IAP. IAP is the automatic sequential workflow for analyzing keratitis, conjunctivitis, and pterygium with smartphone images. IAP Image Analysis Pipeline, IQC Image Quality Controller, DSD Disease Detector, SVA Severity Analyzer.

MOSAIC was designed as an automated and extensible system processing input images and generating output reports (Figure 1). MOSAIC comprises two primary modules: the Agent Allocator and the Image Analysis Pipeline (IAP). The Agent Allocator functions as a router, assigning agents for various sub-tasks within the IAP. These agents are driven by prompt engineering techniques and short-term memory (STM) mechanisms. Prompt engineering has emerged as a crucial method for adapting large language models (LLMs) to specific downstream tasks (Liu et al., 2023). Drawing inspiration from previous studies, we composed a set of instruction prompts (Supplemental Note S1) to “anthropomorphize” MLLMs into distinct agents, enhancing and calibrating them for multiple tasks (Kang and Kim, 2024). The STM was implemented using few-shot prompt learning, a technique that enhanced model performance by providing a small number of examples to the model (Brown et al., 2020).

The IAP of MOSAIC comprises a sequential combination of three agents. First, the “Image Quality Controller” (IQC) assesses the quality of the input image, determining its eligibility for subsequent tasks. If the IQC deems the image “eligible”, it is then forwarded to the “Disease Detector” (DSD) for disease identification. Otherwise, an error message is generated, explaining the reason for ineligibility and providing instructions for capturing an eligible image. Following disease detection, the image is transmitted to the “Severity Analyzer” (SVA) to evaluate the severity of the identified disease. Based on the results from each agent in the IAP, a comprehensive final report is yielded to the user.

### Prompt engineering and STM

Agents in IAP were “anthropomorphized” by a set of identities and memories. These identities were constructed using “instruction prompts” that were engineered in five dimensions: 1) Assigning a name to the agent that the model would perform; 2) Defining the intent and motivation that describe the problem the agent should solve; 3) Specifying the knowledge the agent should possess; 4) Customizing the output format for generation; 5) Establishing guardrails to prevent inappropriate responses (White et al., 2023).

To fully harness the potential of models, we employed STM for agents in IAP using a few-shot prompt learning paradigm (Supplementary Figure S1). We conducted three levels of few-shot prompt learning in this study: zero-shot, one-shot, and five-shot, to observe changes in system performance and determine the optimal level for our system. The images utilized for memory construction were obtained from an independent Jiangdong (JD) clinical center to avoid feature leakage.

### UCSI dataset

MOSAIC was evaluated on the UCSI dataset, which involved ocular surface images captured by various smartphone brands from independent clinical centers. The imaging settings, including zoom scale, exposure, and camera mode, were maintained as the default. For subset A, labels were established through a consensus among three experts, following criteria proposed in our previous study (Li et al., 2021a). In cases of disagreement, a panel of OSD specialists, including a senior specialist with 20 years of clinical experience, convened to deliberate until reaching a unanimous decision. For subsets B, C, and D, image labels were determined by reviewing patients’ medical records and associated media. The definition of mild-stage keratitis adhered to guidelines from previous studies (Stapleton et al., 2012; Keay et al., 2008; Li et al., 2021b). Pterygium grading criteria primarily focused on surgical timing, as indicated by the location of the pterygium head relative to the corneal limbus and pupil (Liu et al., 2024; Maheshwari, 2007).

## Comparison of leading MLLMs

As MLLMs form the backbone of MOSAIC, we conducted comparative analyses of three MLLMs to identify the most suitable model for our system: gpt-4-turbo (GPT4V, OpenAI), claude-3-opus (CLD3O, Anthropic), and gemini-1.5-pro-latest (GM15P, Google). Models were requested through the Python library’s official application program interface (API) to prevent additional data processing between the model and user, which could occur in chatbot web interfaces. Given the inherent stochasticity of transformer-based generative models, we carefully controlled hyperparameters to ensure the reproducibility of results. The detailed settings are provided in Supplementary Table S1. Generated responses were recorded and analyzed to evaluate the system’s performance.

## Image quality control

**FIGURE 2:**
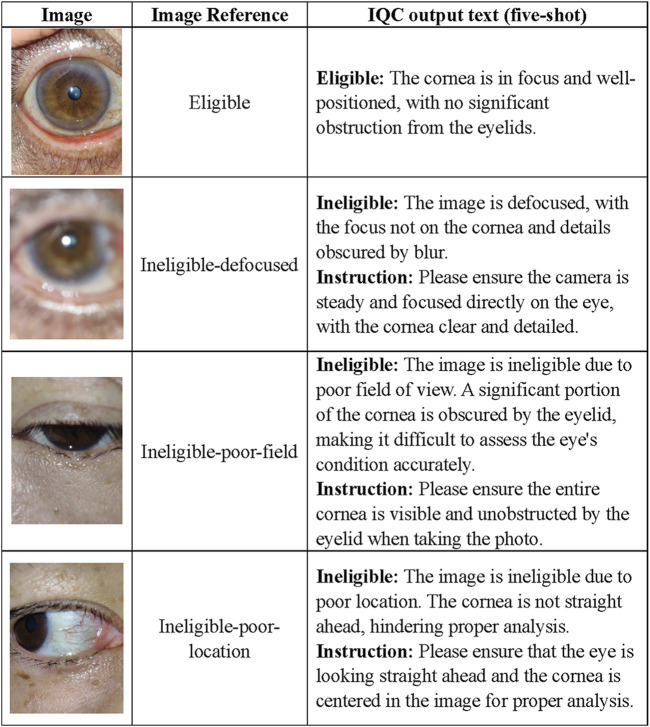
Task examples of the IQC. Based on our previous study on ocular surface image quality, we employed the following definitions for image quality categories. An image is classified as ineligible if it meets any of the following criteria: 1) Defocused images refer to blurry images in which the focus is not on the cornea. 2) Poor-field images refer to images in which one-fifth of the cornea was covered by eyelids. 3) Poor-location images refer to images in which one-fifth of the cornea was blurred because the cornea was not straight ahead. 4) An image quality is deemed eligible if it does not meet any of the aforementioned criteria. IQC Image Quality Controller.

The MOSAIC system was designed for non-professionals who may lack medical imaging experience, enabling them to capture high-quality images using consumer-grade devices in non-clinical settings. To ensure the reliability of subsequent disease-related tasks, we implemented the IQC as the guardian of the IAP. The primary function of the IQC is to classify input images into four categories: eligible, defocused, poor-field, and poor-location. Additionally, the IQC provides a detailed rationale for each classification decision (examples are shown in Figure 2). Only images classified as “eligible” by the IQC will be passed to the DSD. If the IQC deems an image “ineligible”, subsequent tasks will not be performed. Additionally, the system will return a message to the user explaining the reason for ineligibility and recommending effective approaches to capture another image of eligible quality.

### Disease detection

**FIGURE 3:**
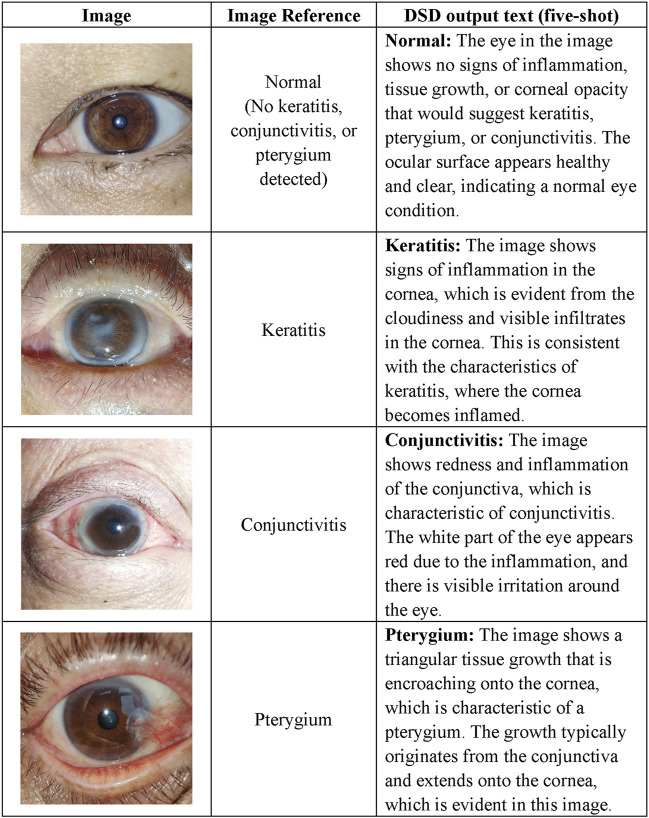
Task examples of the DSD. The diagnostic definitions are as follows. 1) Keratitis: Keratitis is the inflammation of the cornea. 2) Pterygium: Pterygium is a roughly triangular tissue growth extending from the conjunctiva onto the cornea. 3) Conjunctivitis: Conjunctivitis refers to inflammation of the outermost layer of the white part of the eye or the inner surface of the eyelid. 4) Normal: No signs of the aforementioned conditions. DSD Disease Detector.

In this study, we evaluated MOSAIC, focusing on three common OSDs: keratitis, conjunctivitis, and pterygium. After passing through the IQC, the input image will be conveyed to the DSD for disease detection. If the DSD yields “No keratitis, conjunctivitis, or pterygium detected”, consequent severity analysis will not be performed, and MOSAIC will generate a report with negative results for the user. Otherwise, DSD provides a diagnosis based on the image. The OSDs’ definitions and clinical characteristics were incorporated into the DSD identity prompts and STM to promote the alignment of visual and natural language information. Examples of this phase are presented in Figure 3.

### Severity analysis

**FIGURE 4:**
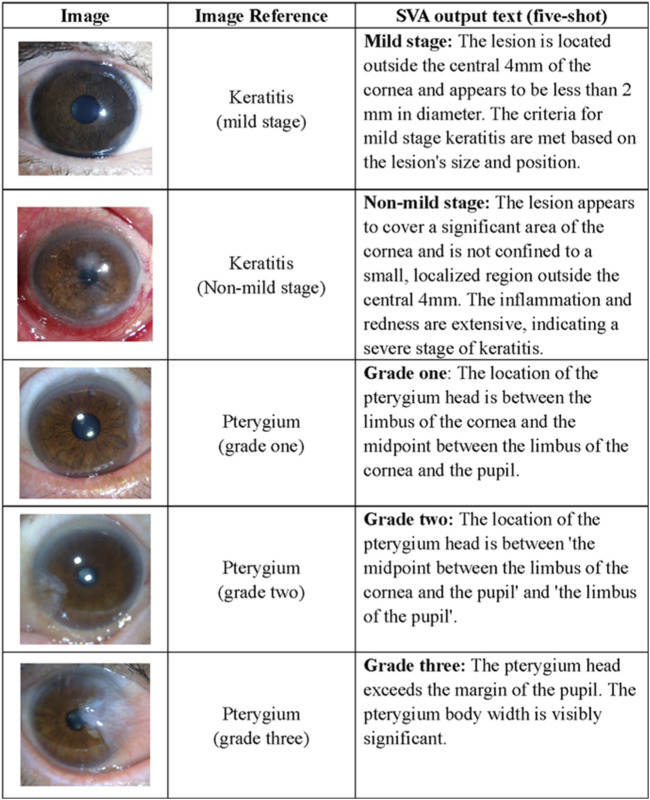
Task examples of the SVA. The definition of keratitis in the mild stage refers to the lesion located outside the central cornea with a diameter of less than 2 mm. The criteria of pterygium grading mainly focus on the surgical timing indicated by the location of the pterygium head, corneal limbus, and pupillary, which categorizes cases as grade one if the length of the limbal invasion is between 0 and 2 mm; as grade two if the invasion is between 2 and 4 mm and as grade three if the invasion was exceeding 4 mm. SVA Severity Analyzer.

Early detection of keratitis, particularly in its mild form when clinical features are subtle, is crucial for optimizing visual outcomes. To address this, we designed a function to detect mild-stage keratitis for the SVA. The definition of mild keratitis aligned with the established criteria for grading keratitis severity, which categorized cases as mild if the lesion was located outside the central 4 mm of the cornea and had a diameter less than 2 mm. For pterygium, surgical intervention is the primary treatment when it encroaches upon the cornea and compromises visual acuity, as the restoration of corneal topography is significantly related to pterygium development. To monitor the pterygium progression and facilitate timely surgical intervention, we incorporated a function for the SVA to grade pterygium severity, following the criteria that mainly considered pterygium size and its relationship to the cornea. Examples of this phase are presented in Figure 4.

### Interpretability of MOSAIC

In contrast to conventional deep learning models for classification tasks that only output labels, MLLMs can provide not only predicted labels but also natural language explanations for their decision-making. To evaluate the interpretative potential of MOSAIC, we calculated and visualized the ROUGE-L (Recall-Oriented Understudy for Gisting Evaluation) F1 score metric between the image description generated by the model and the reference explanations used in prompt engineering. This analysis quantified MOSAIC’s image understanding ability and provided insights into its interpretability.

### Statistical analysis

Statistical analyses were conducted using Python 3.10.14. The differences in accuracy (ACC) were analyzed using the McNemar test. All statistical tests were two-sided with a significance level of 0.05. The interpretability assessment was conducted with the rouge package (pypi.org/project/rouge) and visualized with R 4.4.1 ggplot2 package (ggplot2.tidyverse.org).

### Ethics statement

The study was approved by the Institution Review Board of NEH (identifier, 2020-qtky-017) and adhered to the principles of the Declaration of Helsinki. Informed consent was exempted, due to the retrospective nature of the data acquisition and the use of deidentified images.

## Results

### Dataset characteristics

The UCSI dataset comprised a total of 375 images from 290 eyes in four distinct subsets for various sub-tasks. Subset A comprised 60 images categorized into four image qualification groups: eligible, defocused, poor-field, and poor-location. Subset B consisted of 140 images classified into four diagnostic categories: keratitis, conjunctivitis, pterygium, and normal. Subset C included 70 images divided into two keratitis stage categories: mild stage and non-mild stage. Subset D encompassed 105 images categorized into three pterygium stages: observation (grade one), surgical consideration (grade two), and immediate surgery (grade three). Images collected from Ningbo Eye Hospital (NB) and Eye Hospital of Wenzhou Medical University (WZ) were utilized to evaluate the performance of MOSAIC, while images from JD were employed to construct STMs. Detailed information regarding the datasets is presented in Table 1.

**TABLE 1:** Composition of the UCSI dataset.

Subset	Evaluation task	Test data		Memory construction data (* N-shot)
		NB	WZ	JD
A	Image quality control	20	20	4
B	OSDs detection	81	39	4
C	Keratitis stage analyzing	30	30	2
D	Pterygium stage analyzing	60	30	3

The UCSI, dataset comprises four subsets (A-D) designed for distinct tasks. The memory construction data was sourced exclusively from the JD, center to prevent feature leakage. The N-shot prompt learning provides models with N pairs of examples for each category during the prediction. UCSI, union centers smartphone image; NB, ningbo eye hospital; WZ, eye hospital of wenzhou medical university; JD, Jiangdong Eye Hospital. N number, OSD, ocular surface disease.

### Performance of the IAP components

**FIGURE 5:**
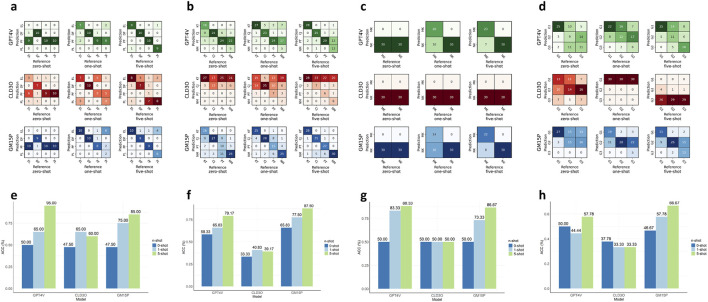
Comparing the performance of MLLMs and few-shot levels for agents in the MOSAIC. **(a–d)**. Confusion matrices describing the prediction results of three MLLMs and three few-shot levels for agents IQC, DSD, SVA (keratitis stage), and SVA (pterygium grade) in order. **(e–h)**. The accuracies of three MLLMs and three few-shot levels for agents in the same order. IQC Image Quality Controller, DSD Diseases Detector, SVA Severity Analyzer. MLLMs multimodal large language model. EL eligible, DF defocused, PF poor-field, PL poor-location. KT keratitis, CJ conjunctivitis, PT pterygium, NM normal, MK keratitis (non-mild stage), NK keratitis (mild stage), G1 (pterygium grade one), G2 (pterygium grade two), G3 (pterygium grade three).

The performance of the IAP components varied depending on the specific models employed and the extent of few-shot prompt learning implemented. Generally, we observed that as the number of learning examples increased, the ACCs of the IAP components demonstrated improving trends (Figure 5; Table 2).

**TABLE 2:** Differences of ACC between few-shot levels.

Sub-tasks		Few-shot level	GPT4V	CLD3O	GM15P
IQC		0 vs 1	<0.01	<0.01	<0.01
		0 vs 5	<0.01	<0.01	<0.01
		1 vs 5	<0.01	<0.01	<0.01
DSD		0 vs 1	<0.01	<0.01	<0.01
		0 vs 5	<0.01	<0.01	<0.01
		1 vs 5	<0.01	<0.01	<0.01
SVA	Keratitis Stage	0 vs 1	<0.01	**0.77**	<0.01
		0 vs 5	<0.01	**0.84**	<0.01
		1 vs 5	<0.01	**0.17**	<0.01
	Pterygium Grade	0 vs 1	<0.01	<0.01	<0.01
		0 vs 5	<0.01	<0.01	<0.01
		1 vs 5	<0.01	**0.51**	<0.01

Overall, the performance of each IAP, component improved as the few-shot level increased. Exceptionally, the performance of the CLD3O model did not improve even in the five-shot setting. ACC, accuracy; IAP image analysis pipeline; IQC, image quality controller; DSD, diseases detector; SVA, severity analyzer, GPT4V gpt-4-turbo, CLD3O claude-3-opus, GM15P gemini-1.5-pro-latest.

The IQC achieved an ACC of 95.00% in assessing the quality of input images utilizing the GPT4V model in the five-shot setting. In the zero-shot setting, the ACC did not exceed 50.00% for any of the tested models. However, with only one-shot prompt learning, the ACC improved to 65.00%–75.00%, indicating the significance of few-shot prompt learning. Additionally, the GM15P model achieved 85.00% ACC with the five-shot setting, while the CLD3O model demonstrated unsatisfactory performance in IQC, with ACC ranging from 47.50% to 65.00%.

Based on images that met quality criteria as screened by IQC, DSD achieved an ACC of 87.50% in detecting keratitis, conjunctivitis, pterygium, and normal utilizing the GM15P model in the five-shot setting, demonstrating superior performance compared to the GPT4V model. The CLD3O model proved unsuitable as the backbone of DSD, with ACC ranging from 33.33% to 40.83%.

With the diagnosis decision made by DSD, SVA further analyzed the severity of the detected disease. For recognizing mild-stage keratitis, SVA achieved an ACC of 88.33% with the GPT4V model in the five-shot setting. It is worth mentioning that all three models only attained the ACC of 50% in the zero-shot setting, classifying all test images as “non-mild stage of keratitis”. The GPT4V model not only demonstrated the best performance among the three in the five-shot setting but also attained an ACC exceeding 80.00% (83.33%) with only one learning example, exhibiting outstanding few-shot prompt learning capability. Remarkably, few-shot prompt learning seemed ineffective for the CLD3O model in this sub-task, considering all images as “non-mild stage keratitis” even when the level of learning increased to five. For grading pterygium, SVA achieved an ACC of 66.67% with the Google model in the five-shot setting. The CLD3O model still demonstrated limited performance of 33.33%–37.78%, and OpenAI attained unsatisfactory results of 50.00%–57.78%.

Based on these results, we employed the optimal model for each sub-task agent with five learning shots, enabling MOSAIC to function as a flexible framework that leverages each model’s unique strengths.

### Interpretability assessment

**FIGURE 6:**
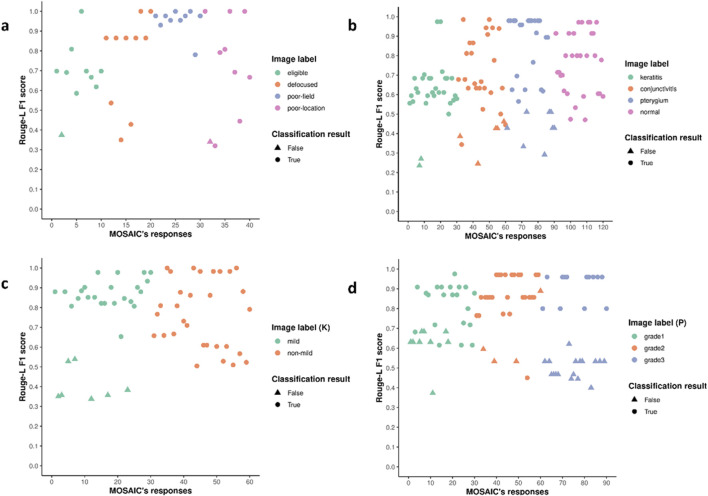
The distribution of ROUGE-L F1 scores for MOSAIC’s interpretation of images. **(a–d)**. The ROUGE-L F1 scores of each test image for IQC, DSD, SVA (keratitis stage), and SVA (pterygium grade). Higher scores are aligned with correct classification results, and lower scores are aligned with wrong classification results, suggesting that the decisions made by MOSAIC agree with the reasonings. IQC Image Quality Controller, DSD Diseases Detector, SVA Severity Analyzer. (K) Keratitis stages, (P) pterygium grades. ROUGE-L Recall-Oriented Understudy for Gisting Evaluation.

ROUGE-L F1 scores were employed in this study to quantify MOSAIC’s image understanding capability. The IAP components attained the average ROUGE-L F1 score of 0.78, 0.70, 0.72, and 0.76 for IQC, DSD, SVA (detecting mild stage keratitis), and SVA (grading pterygium), respectively. Figures 6a–d illustrates the distribution of the ROUGE-L F1 scores for the system’s comprehension processes. Correctly classified test images demonstrated high scores, indicating that accurate classification decisions were based on proper interpretations of the input images. Conversely, misclassified test images exhibited lower scores in the system’s comprehension processes, suggesting that inadequate image understanding led to classification errors.

## Discussion

In this study, we developed MOSAIC, an MLLM-based AI agent system for detecting three common OSDs from smartphone images. We evaluated the system using images from two independent clinical centers within the UCSI dataset. Three MLLMs were assessed leveraging various levels of few-shot prompt learning. Additionally, we quantified the image understanding capability of the MLLMs to interpret the reasoning underlying their decision-making processes. With only five-shot learning examples, MOSAIC achieved an ACC of 95.00% in controlling input image quality (with GPT4V model), 87.50% in detecting three OSDs (with GM15P model), 88.33% in recognizing mild-stage keratitis (with GPT4V model), and 66.67% in determining the progression stage of pterygium (with GM15P model).

OSDs can lead to severe consequences if not addressed promptly, especially in less developed communities where specialized equipment and experts are scarce. Patients typically seek treatment only after their visual acuity has been significantly compromised (Burton, 2009). MOSAIC can enable patients to utilize AI models as personal healthcare copilots. Users can simply capture an ocular surface image with a smartphone, upload it to the system, and receive a comprehensive report. Through this approach, MOSAIC demonstrates promise in empowering high-risk populations to proactively manage their eye health from home, for example, detecting keratitis at an early stage before clinical features become apparent or monitoring the progression of pterygium to determine optimal surgical timing to reduce the risk of vision impairment.

To identify the optimal model for each agent in MOSAIC, we evaluated three MLLMs–GPT4V, CLD3O, and GM15P—across subtasks within the IAP module. We found that: 1) The GPT4V model surpassed the other models in determining the input image quality and detecting mild stage keratitis. 2) The GM15P overcame the other models in detecting three OSDs from the normal and grading pterygium progression (Table 3). The varying performance of different models across subtasks may be attributed to differences in their training data and model architectures. Notably, in identifying keratitis severity, all three models demonstrated 100.00% sensitivity for “non-mild stage keratitis” in the zero-shot setting, suggesting the models’ conservative approaches when faced with potentially high-stakes tasks without prior examples. In this context, the GPT4V model significantly improved its ACC to 83.33% with just one-shot learning, demonstrating outstanding few-shot prompt learning capabilities. In contrast, the CLD3O model consistently underperformed in this study, contradicting reported claims of its excellency (Kaczmarczyk et al., 2024; Toufiq et al., 2023; Shojaee-Mend et al., 2024). This discrepancy underscores the importance of evaluating models comprehensively across multiple modalities and dimensions.

**TABLE 3:** Differences of ACC between models.

Sub-tasks		MLLM	Zero-shot	One-shot	Five-shot
IQC		GPT4V vs CLD3O	<0.01	**0.44**	<0.01
		GPT4V vs GM15P	<0.01	<0.01	<0.01
		CLD3O vs GM15P	**0.41**	<0.01	<0.01
DSD		GPT4V vs CLD3O	<0.01	<0.01	<0.01
		GPT4V vs GM15P	<0.01	<0.01	<0.01
		CLD3O vs GM15P	<0.01	<0.01	<0.01
SVA	Keratitis Stage	GPT4V vs CLD3O	**0.12**	<0.01	<0.01
		GPT4V vs GM15P	**0.47**	<0.01	<0.01
		CLD3O vs GM15P	**0.90**	<0.01	<0.01
	Pterygium Grade	GPT4V vs CLD3O	<0.01	<0.01	<0.01
		GPT4V vs GM15P	<0.01	<0.01	<0.01
		CLD3O vs GM15P	<0.01	<0.01	<0.01

In the zero-shot setting, three MLLMs, performed comparably in detecting the keratitis stage. As the few-shot level increased, the differences in the models’ learning capabilities emerged. ACC, accuracy; IQC, image quality controller; DSD, diseases detector; SVA, severity analyzer, GPT4V gpt-4-turbo, CLD3O claude-3-opus, GM15P gemini-1.5-pro-latest, MLLM, multimodal large language model.

To enhance model performance and reduce training data costs, fine-tuning pre-trained foundation models for specific medical downstream tasks has become a common development paradigm (Tajbakhsh et al., 2016). However, while fine-tuned models demonstrate improved performance in particular domains, their generalization capability in other domains inevitably declines. Moreover, fine-tuning requires high-powered devices, experienced engineers, and domain-specific data, which are often inaccessible in less developed areas. In this study, we aligned MLLMs with diverse downstream tasks by constructing and injecting task-specific memories into the model’s “thinking” process. This approach, termed few-shot prompt learning, eliminates the need for extensive computational resources or large datasets. Instead, it requires only a small set of images and text instructions to construct the necessary memory, enabling the model to achieve impressive performance across various medical tasks.

Despite their remarkable performance capabilities, large language models remain susceptible to spontaneous hallucinations, which significantly compromises their reliability and trustworthiness. To enhance the MOSAIC’s credibility, we instructed the models to generate both a predicted label (e.g., “image with eligible quality”, “keratitis”, “pterygium grade two”, etc.) and an explanation for the decision-making process, including the interpretation of the test image. Through this approach, MOSAIC can provide suggestions for a patient’s ocular surface health conditions using eye images, effectively serving as a personal health copilot. To quantify the system’s interpretability, we calculated the ROUGE-L F1 scores of the generated explanations. The distribution plot of these scores revealed that correctly classified images exhibited higher scores, while misclassified images demonstrated lower scores. This feature provides users with an additional safeguard to model hallucination, as the higher the score, the more reliable the information the system offers.

Recently, several studies exploring the border of MLLMs in clinical scenarios have been published. Kaczmarczyk et al. evaluated the accuracy and responsiveness of MLLMs in answering the NEJM Image Challenge dataset and found that the best model demonstrated an accuracy of 58.8%–59.8% among various models (Kaczmarczyk et al., 2024). They also found that a model may refuse to answer some questions, which also happened in our preliminary experiments and was solved by the strict engineering of prompts (Table 4). Zhu et al. evaluated the performance of MLLM in interpreting radiological images and formulating treatment plans, finding that it achieved 77.01% accuracy on the United States Medical Licensing Examination questions (Lingxuan et al., 2024). Compared to prior studies, our research had several significant features. Firstly, we designed an AI agent system with extensible components to deal with queries about OSDs in non-clinical environments automatically. Models do not just act as a “black box” to handle inputs and yield outputs in our study. Instead, it was utilized as a backbone “engine” for each step in the whole system. The architecture of MOSAIC could be transferred to other similar research, and the agents allocated by the Agent Allocator can be extended according to the study purposes. Secondly, given the inevitable randomness of the transformer-based model, we controlled the hyper-parameters of involved models, including “temperature”, “max-token”, etc., to make our results reproducible. Last, the images in this study were not disclosed before, eliminating the possibility of dataset contamination, wherein the test images might have been inadvertently included in the models’ training datasets.

**TABLE 4:** Responsiveness in preliminary experiments without prompt engineering.

Agent	Image label	Responsiveness in the zero-shot setting	Responsiveness in the five-shot setting
IQC	-	100.00%	100.00%
**DSD**	Keratitis	85.71%	96.77%
	Conjunctivitis	83.33%	100.00%
	Pterygium	68.18%	93.75%
	Normal	63.83%	71.43%
SVA	-	100.00%	100.00%

In our preliminary experiments, we observed that models occasionally refuse to respond in some cases. After careful refinement of instruction prompts, the responsiveness increased to 100.00%, demonstrating the critical role of prompt engineering in optimizing interactions with MLLMs. IQC, image quality controller; DSD, diseases detector; SVA, severity analyzer; MLLM, multimodal large language model.

This study has several limitations. First, we evaluated MOSAIC, which primarily focused on OSDs and did not extend to other diseases. This narrow focus, while allowing for a detailed analysis of OSDs, limits the applicability of our findings to a broader range of eye conditions. We intend to expand our evaluation to other diseases in future studies. Second, the study was conducted on a relatively limited dataset. While test data contained images from two individual centers, it might not fully represent the diversity of real-world scenarios. In future work, we plan to curate more extensive and diverse datasets to validate further and potentially enhance the robustness of our system. Third, our study concentrated on the three leading proprietary models, excluding open-source alternatives from the assessment. However, our intention was to enhance AI accessibility globally, particularly in underdeveloped regions, while deploying open-source MLLMs requires substantial computational resources. Moreover, proprietary models currently offer greater accessibility and user-friendliness through their APIs.

## Conclusion

In conclusion, we developed MOSAIC, an MLLM-based AI agent system for detecting and grading common OSDs using smartphone images. Leveraging MLLMs, prompt engineering, and few-shot prompt learning, MOSAIC demonstrated remarkable performance in image quality control, disease detection, and severity analysis. This system shows potential for improving early detection and management of OSDs in non-clinical settings, particularly in resource-limited areas.

## Data Availability

The raw data supporting the conclusions of this article will be made available by the authors, without undue reservation.

## Abbreviations

OSD, Ocular surface disease; AI, artificial intelligence; MOSAIC, Multimodal Ocular Surface Assessment Intelligent Copilot; MLLM, multimodal large language model; UCSI, Union Centers Smartphone Image; IAP, Image Analysis Pipeline; STM, short-term memory; IQC, Image Quality Controller; DSD, Disease Detector; SVA, Severity Analyzer; JD, Jiangdong; GPT4V, gpt-4-turbo; CLD3O, claude-3-opus; GM15P, gemini-1.5-pro-latest; API, application program interface; ROUGE-L, Recall-Oriented Understudy for Gisting Evaluation; ACC, accuracy; NB, Ningbo Eye Hospital; WZ, Eye Hospital of Wenzhou Medical University.
